# The effects of motivational self-care promotion on depressive symptoms among adults with type 2 diabetes: A systematic review and *meta*-analysis

**DOI:** 10.1016/j.pmedr.2023.102431

**Published:** 2023-09-20

**Authors:** Ulla Mikkonen, Ari Voutilainen, Tuomas Mikola, Johanna Roponen, Sanna Rajapolvi, Soili M. Lehto, Anu Ruusunen, Pekka Mäntyselkä

**Affiliations:** a Institute Of Public Health and Clinical Nutrition, University Of Eastern Finland, P.O. Box 1627, FI-70211 Kuopio, Finland; b Primary Health Care Center, Wellbeing Services County of North Savo, P.O. Box 1711, FI-70211 Kuopio, Finland; c Institute Of Clinical Medicine, University Of Eastern Finland, P.O. Box 1627, FI-70211 Kuopio, Finland; d Institute Of Clinical Medicine, University Of Oslo, P.O. Box 1171 - Blindern, 0318 Oslo, Norway; e R&D Department, Division Of Mental Health Services, Akershus University Hospital, 1478 Lørenskog, Norway; f Department Of Psychiatry, University Of Helsinki, P.O. Box 22, FI-00014 University Of Helsinki, Finland; g Department Of Psychiatry, Kuopio University Hospital, Wellbeing Services County of North Savo, P.O. Box 1711, FI-70211 Kuopio, Finland; h Deakin University, Institute For Mental And Physical Health And Clinical Translation (IMPACT), Food & Mood Centre, School Of Medicine, Barwon Health, P.O. Box 281 Geelong, Victoria 3220, Australia; i Clinical Research And Trials Centre, Kuopio University Hospital, Wellbeing Services County Of North Savo, P.O. Box 1711, FI-70211 Kuopio, Finland

**Keywords:** Type 2 Diabetes, Depression, Health Behavior, Self Care, Motivational Interviewing, Meta-Analysis, Systematic Review

## Abstract

In individuals with type 2 diabetes (T2D), comorbid depression leads to increased health care costs and unsatisfactory treatment outcomes. Supporting healthy behaviors and self-efficacy might provide means to prevent depressive symptoms. We assessed the effects of motivational interviewing (MI) – based self-care promotion that specifically targets health behaviors, on depressive symptoms in adults with T2D. We followed PRISMA guidelines and searched Pubmed, Scopus, PsycINFO, Cinahl, and Cochrane Library to find randomized controlled trials (RCTs) published up to February 2023. Eligible RCTs had to target the T2D adult population, examine MI-based interventions that focus on multiple health behaviors, and measure depressive symptoms on a validated scale. Standardized mean differences (SMD) with 95% confidence intervals were calculated using a random-effects model. We used the Grading of Recommendations Assessment, Development and Evaluation (GRADE) approach to assess the certainty of the evidence. After the screening, eleven studies with 2,682 individuals were eligible for the narrative synthesis. A *meta*-analysis of nine studies favored interventions with a pooled SMD of −0.19 (95% Cl = -0.34 to −0.05, p = 0.008, I^2^ = 52%). Due to the indirectness and imprecision of the evidence, we assessed the certainty of evidence based on GRADE as low. MI-based self-care promotion with a focus on health behaviors and implemented by a well MI-trained person had a preventive effect on depressive symptoms among adults with T2D. However, the certainty of evidence remained low. In future trials, the effect of MI-based self-care promotion on depression should be studied in clinically depressed populations.

## Highlights

• Supporting self-care in type 2 diabetes may offer means to prevent depressive symptoms.
• Improving health behaviors has a positive effect on depressive symptoms.
• Motivational interviewing provides tools for supporting the key elements of self-care.

## 1 Introduction

The relationship between depression and type 2 diabetes (T2D) is bidirectional and possible pathways are biological, cognitive, and behavioral (Anderson et al., 2001, Golden et al., 2008). In general, depressed mood leads to an increased risk of T2D (Mezuk et al., 2008, Rotella and Mannucci, 2013), and factors explaining the association may include non-adherence to self-care, medical appointments, or medication (Gonzalez et al., 2008), altered inflammatory pathways (Nguyen et al., 2021, Wang et al., 2021), microvascular dysfunction (van Agtmaal et al., 2017), unhealthy behaviors, and obesity (Golden et al., 2008). Correspondingly, T2D predisposes a person to depression (Mezuk et al., 2008), and the pathways are partially the same as those described above, but the impact of T2D is also explained by psychological stress and the psychological burden of having been diagnosed with diabetes (Golden et al., 2008, Knol et al., 2007, Nouwen et al., 2011). Furthermore, among depressed people with T2D, the risk of diabetes complications is significantly increased (Nouwen et al., 2019) and, similarly, comorbid depression among people with T2D is associated with increased mortality (Park et al., 2013). Altogether, the presence of comorbid depression in individuals with T2D leads to increased use of health services (Tusa et al., 2019, Himelhoch et al., 2004), diabetes complications (Nouwen et al., 2019) and to increased health care costs (Egede et al., 2002). Therefore, from the perspectives of both patients and health care providers, it is highly important to find ways to reduce depressive symptoms in T2D populations.

Among depressed individuals, unhealthy behaviors such as physical inactivity, unhealthy diet and smoking are common, and play a role in the development and management of depression (Jacka and Berk, 2013, Sarris et al., 2014, Sarris et al., 2020). Furthermore, interventions that target health behaviors can be applied as a supportive treatment for depression (Firth et al., 2019, Kvam et al., 2016, Riper et al., 2014). However, unhealthy behaviors are often related, which is why focusing on only one of them might be insufficient. Thus, there is an increasing interest to examine interventions that target more than one health behavior simultaneously to reduce depressive symptoms. Some evidence already exists of the effects of these multicomponent lifestyle interventions on depressive symptoms among heterogeneous populations (Gómez-Gómez et al., 2020, Wong et al., 2021, Cezaretto et al., 2016). However, none of the previous reviews have focused on motivational, health behavior-oriented self-care promotion among individuals with T2D.

The treatment as usual (TAU) for T2D includes multicomponent health promotion at different stages of the treatment procedure and it aims at supporting the patient's self-care, such as adherence to health behavior changes. Indeed, self-care plays a crucial role in the treatment of T2D (Beck et al., 2017). Supporting patients for better self-care is also valuable from a psychological point of view, since an active role in self-care is associated with higher quality of life (Wang et al., 2017, Otsu and Moriyama, 2011). Thus, it is worth asking whether more effective self-care promotion could help to prevent or influence depressive symptoms among individuals with T2D. It should be noted that self-care might be burdensome, which is why patients’ self-efficacy should be supported simultaneously (Young-Hyman et al., 2016, Bandura, 1997). Self-efficacy is defined as an individual's confidence in their ability to perform well (Bandura, 1997). Self-efficacy has been related to improvements in self-care behaviors, such as diet and exercise (King et al., 2010). In addition, higher self-efficacy has been associated with fewer depressive symptoms and higher quality of life (Peters et al., 2019).

Nevertheless, health behavior changes often require a high level of motivation. Motivational interviewing (MI) is a patient-centered, nonauthoritarian approach for encouraging patients to find motivation and commitment to change. In 1983, Miller described the first principles of MI (Miller, 1983), and, in the 1990 s, Miller and Rollnick (Miller and Rollnick, 1991) further developed the concept and published more specific strategies and counseling structures to be applied in clinical practice. Expressing empathy, supporting self-efficacy, avoiding argumentation, rolling with patient’s resistance, and developing discrepancy are the basic principles of MI. In addition, hearing the patient’s perspectives, ideas, and experiences through open-ended questions; building the patient’s confidence in their ability to change; and summarizing the key points made by a patient are the core skills in facilitating communication between the patient and MI practitioners (Miller, 1983). However, conducting MI requires thorough training (Schwalbe et al., 2014) but is applicable in the clinical setting. Various psychotherapies are well known to be effective in treating depressive symptoms, but self-care promotion in T2D is mostly performed by nurses and dietitians. Therefore, it is important to examine the potential impact of MI-based self-care promotion on depressive symptoms when it is carried out by health care professionals other than therapists.

To summarize, health behavior-oriented self-care promotion for T2D patients may provide means to influence depressive symptoms, and MI aims to support self-efficacy and motivation for health behavior changes. Therefore, the objective of this systematic review and *meta*-analysis was to assess the effects of MI-based health behavior-oriented self-care promotion on depressive symptoms in adults with T2D compared with TAU, wait-list, non-active controls, or attention controls.

## 2 Materials and methods

### 2.1 Search strategy

This study followed the Preferred Reporting Items for the Systematic Reviews and Meta-analyses (PRISMA) guidelines (Page et al., 2021) (Supplemental eTable 1 presents the PRISMA Checklist). The protocol was established in the International Prospective Register of Systematic Reviews prior to conducting the review (ID**:** CRD42021249399]. An information specialist created the search strategy according to the components of PICO (Supplemental eTable 2 demonstrates the search strategies for all databases). To produce the most reliable evidence, only randomized controlled trials (RCTs) were included (Sackett, 1989). The language of publication was restricted to English, as this restriction appears not to affect the conclusion of the systematic review and *meta*-analysis (Morrison et al., 2012). Pubmed/MEDLINE, Scopus, PsycINFO, Cinahl, and Cochrane Library (CENTRAL) were searched. The primary search was conducted in April 2021, and the re-search in February 2023, to identify recently published studies for eligibility. Full text peer-reviewed articles were included. No limitations were constructed for publication dates. Cochrane Reviews was searched for *meta*-analyses related to the topic and the reference lists of those were reviewed to identify relevant studies. As well, all potential trials retrieved from CENTRAL were investigated to identify eligible studies. Ethical approval was not required for this systematic review.

### 2.2 Eligibility criteria, study selection, and data extraction

We followed the following inclusion criteria for each PICO element: 1) Population: Adults (≥18 years of age) with T2D, in all health care settings without regional, ethnic, or sex restrictions. Populations with schizophrenia spectrum and other psychotic disorders, bipolar and related disorders, eating disorders, or neurocognitive disorders were excluded because it was our assessment that their symptomatology would potentially cause bias in the measurement of depressive symptoms; 2) Intervention: Multicomponent lifestyle interventions conducted by MI as the only or supportive treatment for T2D. We were specifically interested in health behavior improvements, which is why the interventions had to focus on health behavior change. Interventions had to target health behavior change in physical activity, diet, sleep, stress-management, smoking, and combine two or more of them. MI was considered present if the authors reported in their article that MI was used as a counseling method, and the use of MI was characterized as described by Miller and Rollnick [i.e., interventions have a clear focus on behavioral change and are conducted in accordance with the basic principles of MI (described in the introduction)] (Miller and Rollnick, 1991); 3) Comparator: TAU, wait-list, non-active controls, or attention controls; 4) Outcome: A change in depressive symptoms from baseline to the latest follow-up point measured by validated scales.

Three authors (UM, SR, AR) independently screened titles, abstracts, and full texts in duplicate. Disagreements were resolved by consensus. Cochrane’s screening and data extraction tool Covidence was used to assist in data management (Covidence systematic review software, 2023). Data extraction was performed in duplicate and independently across three authors (UM, JR, TM). The authors, year of publication, country, aims, methods, participants, details of intervention and control arms, outcomes, and notes on funding and conflicts of interest were extracted. In the event of missing data, we attempted to contact the corresponding author of the study in question.

### 2.3 Quality assessment

The risk of bias assessment was done in duplicate and independently across three authors (UM, JR, TM). We used version 2 of Cochrane’s Risk of bias tool, in which the risk of bias is assessed at the outcome level, and rated the risk as “low”, “some concerns” or “high” (Sterne et al., 2019). High risk studies were planned to be excluded from the *meta*-analysis. The Grading of Recommendations Assessment, Development and Evaluation (GRADE) approach was used to assess the certainty of evidence (Balshem et al., 2011). GRADE is a quality assessment tool that guides the authors in summarizing the certainty of evidence by evaluating the study strengths and limitations in different areas (risk of bias, publication bias, inconsistency, indirectness, and imprecision). GRADE assessment was done in duplicate and independently by two authors (UM, AV). They resolved disagreements by consensus.

### 2.4 Data analysis

The primary outcome was a change in depressive symptoms. We calculated mean changes and standard deviations of the means for treatment and control groups in each study based on the study arm means and standard deviations at baseline and follow-up points and conducted the *meta*-analysis in R (R Core Team, 2020) using the “meta” package (Schwarzer et al., 2015). Since the outcome was measured with different scales, we used standardized mean differences (SMD) with 95% confidence interval (CI) as a summary statistic. Heterogeneity was expected to occur across study populations, which is why we used a random-effects model to produce the pooled weighted effect size and P-values. If there were more than one comparator group in a study, we chose the group most similar to TAU as a comparator. An effect size favoring treatment, i.e., a greater reduction in depressive scores, was presented as a negative value in the *meta*-analysis. Statistical heterogeneity across studies was assessed by interpreting confidence intervals, the Cochrane’s Q-test, and I^2^. If I^2^ was more than 75%, we assumed the proportion of true heterogeneity was considerable (Higgins et al., 2022). Publication bias was assessed through visual inspection of funnel plots and executing the Egger’s regression test (Egger et al., 1997).

At the protocol stage, we had a plan to conduct a subgroup analysis according to populations’ depression status. The original studies that required elevated depressive symptoms as inclusion criteria were classified as “depressed” and studies that did not require elevated depressive symptoms for the study entry were classified as “no depression requirement”. In the data extraction, we collected study characteristics that we supposed would potentially have an impact on the effect size of the interventions. First, we performed a random-effects *meta*-analysis with the conventional inverse variance method for study weights and the restricted maximum-likelihood estimator for tau^2^. Second, we carried out preliminary *meta*-regressions in relation to the following study characteristics: the type of RCT, setting, the type of intervention, control, depression scale, the duration of intervention, the delay between the end of intervention and depression assessment, and the depression status at baseline. The purpose of these *meta*-regressions was to recognize meaningful grouping factors for a subgroup *meta*-analysis. Depression status was the only study characteristic that statistically significantly affected the results of preliminary *meta*-regressions (p = 0.021). Third, based on the preliminary *meta*-regressions and a pre-specified plan, we conducted a subgroup *meta*-analysis with respect to the depression status (populations with elevated depressive symptoms versus non-depressed populations at baseline).

## 3 Results

### 3.1 Review

**Fig. 1:**
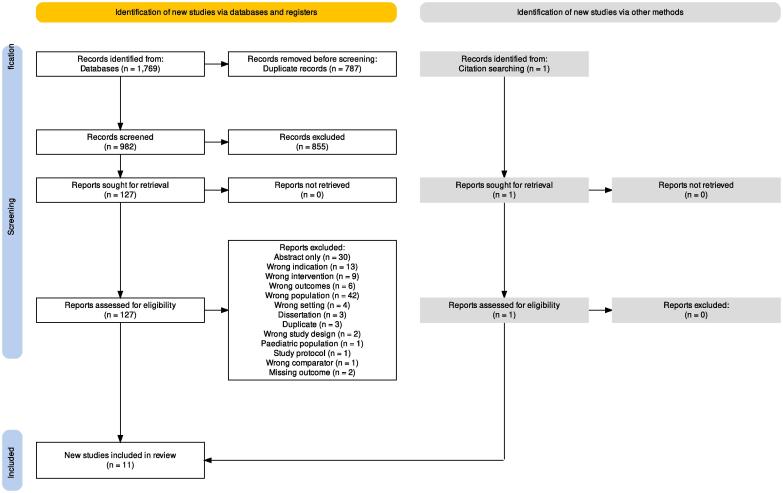
Prisma FLOW Chart presents the study selection process.

We identified 1,770 records with our search strategy. After duplicate removal, 983 records were screened and 855 were judged as irrelevant (Fig. 1). The remaining 128 were screened in full text and 117 of those were ineligible and excluded. Means and standard deviations for depressive symptoms were not reported at baseline and follow-up in three otherwise eligible studies. One of them provided requested estimates after the corresponding author was contacted (Holmen et al., 2014) and it was included. We did not obtain outcome variables for the present *meta*-analysis from the other two studies and, therefore, the studies were excluded (Supplemental eTable3 describes the details of excluded eligible studies). In the end, eleven studies with 2,682 individuals were eligible for the synthesis (Holmen et al., 2014, Ali et al., 2020, Azami et al., 2018, Döbler et al., 2018, Gabbay et al., 2013, Glasgow et al., 2006, Huang et al., 2016, Katon et al., 2010, Swoboda et al., 2017, van der Wulp et al., 2012, Young et al., 2020). Fig. 1 presents the PRISMA Flow diagram (Page et al.,, Haddaway et al., 2022).

The included studies were published between 2006 and 2020 (Table 1). The sample size per study varied between 60 and 545. Participants’ mean age ranged from 52 to 64 years. The details of the included studies are described in Table 1. In addition, Supplemental eTable 4 describes the details of interventions and MI training.

**Table 1:** Characteristics of the included studies for the systematic review.

AuthorsYear (Country)	Population	Design Setting	Age, mean	n, Female%	Intervention Lifestyles Implementation Deliverer	Control	Follow-up (months)	Scale
Glasgow et al.2006 (USA)	≥ 25 years, type 2 diabetes	2-arm RCT, Primary health care	61.5	400, 50.2%	PA + Diet CD-ROM program, In-person session, Phone session, Educational material, Tailored newsletter Health couch	TAU (and computer assisted health risk appraisal)	2	PHQ-9, (0–27)
Katon et al.2010 (USA)	18–80 years; diabetes, coronary heart disease or both; poor disease control within the previous 12 months; PHQ-9 ≥ 10	2-arm RCT, Primary health care	57.4 (intervention), 56.3 (control)	214, 48% (intervention), 56% (control)	General health (self-care activities e.g., exercise) and Collaborative care TAU Educational material, In-person sessions, Phone sessions Nurse (MI) and Primary care physician (Medical care) Nurses received supervision from a psychiatrist, physician and psychologist	Enhanced TAU	12	SCL-20 (0–4)
Van der Wulp et al.2012 (Netherlands)	Type 2 diabetes	2-arm RCT, Primary health care	61 (median)	133, 45.4%	PA + Diet and TAU In-person sessions, Phone sessions Peer (Expert patient)	TAU	3	CES-D (0–60)
Gabbay et al.2013 (USA)	18–75 years, type 2 diabetes, and one or more of the following: (1) HbA1c > 8.5% (69 mmol/mol), (2) blood pressure > 140/90 mmHg, or (3) LDL > 130 mg/dL	2-arm RCT, Primary health care	58 (intervention), 58 (control)	545, 62% (intervention), 55% (control)	General health (lifestyle behavior relevant to T2D management) and TAU In-person sessions Nurse case manager	TAU	24	CES-D (0–60)
Holmen et al.2014 (Norway)	≥ 18 years, type 2 diabetes, HbA1C ≥ 7.1% (54 mmol/mol)	3-arm RCT, Primary health care	57	151, 41%	PA + Diet and TAU Mobile phone apps (Few-Touch Application system) , Phone sessions Diabetes specialist nurse (supervision from clinical psychologist and dietitian)	Few Touch Application + TAU TAU	12	CES-D (0–60)
Huang et al.2016 (Taiwan)	≥ 20 years, type 2 diabetes, CES-D ≥ 16	2-arm RCT, Endocrinology and metabolism outpatient department	56.4	65, 52.5%	Stress management, lifestyle behavior change and dietary education and TAU Groups-sessions (MET + CBT) Research team (clinical nurse and psychotherapist)	TAU	3	CES-D (0–60)
Swoboda et al.2017 (USA)	40–75 years, type 2 diabetes, overweight or obese, and additional risk factor for cardiovascular disease	3-arm RCT, Urban community	56.76 (intervention), 55.41 (control)	60, 67.6% (intervention), 70.6% (control)	PA + Diet and TAU Educational material, In-person session, Phone sessions Registered dietitian	AC	4	PHQ-8 (0–24)
Azami et al.2018 (Iran)	≥ 18 years, type 2 diabetes, HbA1C ≥ 8% (64 mmol/mol)	2-arm RCT, Outpatient endocrine clinic	54.2	142, 65.5%	PA + Diet and TAU Educational material, Group sessions, Phone sessions Diabetes specialist nurse	TAU	6	CES-D Short (0–30)
Döbler et al.2018 (Germany)	18–70 years, type 2 diabetes	2-arm RCT, Rehabilitation center specialized in diabetes care	52	249, 30%	PA, Diet, Smoking, and TAU Educational material, In-person session, Phone sessions Nonmedical dietitian	TAU	12	PHQ-9, (0–27
Ali et al.2020 (India)	≥ 35 years, type 2 diabetes, depressive symptoms (PHQ-9 ≥ 10), at least 1 poorly controlled cardiometabolic parameter	2-arm RCT, Diabetes clinics	52.7	404, 59.2%	Exercise, Diet, Smoking, and Collaborative care TAU In-person or phone sessions Non-physician care coordinator with a background in allied health fields (eg,nutritional counseling, social work) (MI), diabetes physicians (medical care) , supervised by a psychiatrist and diabetologist	TAU	24	SCL-20 (0–4)
Young et al.2020 (USA)	≥ 18 years, type 2 diabetes, HbA1C ≥ 6.5% (48 mmol/mol)	2-arm RCT, Primary health care	59.07	319, 47.3%	General health(PA, nutrition, stress reduction, alcohol, sleep) and TAU Educational material, Mobile-phone apps, Wearable tracking devices, In-person session, Phone sessions Nurse	TAU	9	PHQ-9 (0–27)

Abbreviations: T2D, type 2 Diabetes; RCT, randomized controlled trial; PA, physical activity; TAU, treatment as usual; PHQ, Patient health questionnaire; SCL-20, 20-Item Symptoms Checklist Depression Scale; CES-D, the Center for Epidemiologic Studies Depression Scale; HbA1c, glycosylated hemoglobin; MI, motivational interviewing; MET; motivational enhancement therapy; CBT, cognitive behavioral therapy; AC, attention control; LDL, low-density lipoprotein.

MI-based self-care promotions were provided by non-physician care coordinators with a background in allied health fields (e.g., nutritional counselling or social work) (Ali et al., 2020), diabetes specialist nurses (Holmen et al., 2014, Azami et al., 2018), nurses (Gabbay et al., 2013, Katon et al., 2010, Young et al., 2020), health coaches with varying backgrounds (Glasgow et al., 2006), nonmedical dietitians (Döbler et al., 2018), a clinical nurse and a psychotherapist (Huang et al., 2016), registered dietitians (Swoboda et al., 2017), or peer expert patients (van der Wulp et al., 2012). There were differences in the contents of interventions (Supplemental eTable 4). Most of the studies had a specific goal to support health behavior change but also to support self-care (e.g., blood glucose monitoring, foot care, taking medications), whereas two studies focused only on health behavior change (Döbler et al., 2018, Swoboda et al., 2017). The target health behaviors were physical activity, diet, and smoking (Ali et al., 2020, Döbler et al., 2018), physical activity and diet (Holmen et al., 2014, Azami et al., 2018, Glasgow et al., 2006, Swoboda et al., 2017, van der Wulp et al., 2012), stress management, lifestyle behavior change and dietary education (Huang et al., 2016), general health (Gabbay et al., 2013, Katon et al., 2010), or multiple health behaviors (Young et al., 2020). The intervention groups also received TAU in most of the studies. The duration of interventions varied from 2 to 24 months. In one study (Huang et al., 2016), the group treatment program consisted of four sessions of MI-based motivational enhancement therapy followed by eight sessions of cognitive behavioral therapy; thus, the method of intervention differed from other studies. In addition, the study in question was the only one that included stress management and a psychotherapist as a counsellor. TAU was a comparator in 10 studies, and attention control in one. In three studies (Ali et al., 2020, Huang et al., 2016, Katon et al., 2010), the trial inclusion criteria included depressive symptoms above the validated scale cut-off point. (Table 1).

### 3.2 Risk of bias

The overall risk of bias for all the studies was considered as “some concerns”, except for one with “high risk” (Gabbay et al., 2013) (Supplemental eFigure 1 presents the risk of bias across studies). Most of the studies were judged as “low risk” in Domain 1 (Bias due to randomization), Domain 2 (Bias due to deviations from intended interventions), and Domain 3 (Bias due to missing outcome). All studies were judged as “some concerns” in Domain 4 (Bias due to measurement of the outcome). The basis of the judgment in Domain 4 was that patients were aware of the intervention they received. Since depressive symptoms are a patient-reported outcome, the awareness of intervention may have caused bias in the evaluation of the outcome. Altogether, only two studies were judged as “low risk” in Domain 5 (Bias due to selection of the reported results). The basis of judgement for the rest in Domain 5 was that a pre-specified statistical analysis plan was not published beforehand. We also searched for information on the funding received by the authors of the studies (Supplemental eTable 4).

### 3.3 Meta-analysis

#### 3.3.1 Effect of MI-based self-care promotion on depressive symptoms

**Fig. 2:**
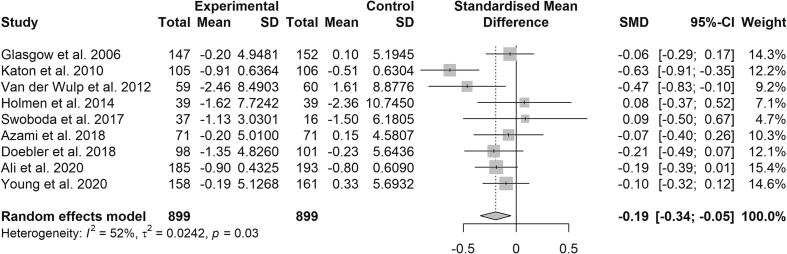
Forest plot for 9 studies combined presents the effect of motivational self-care promotion on depressive symptoms among adults with type 2 diabetes.

The *meta*-analysis of all included studies (k = 10), except for Gabbay et al. (Gabbay et al., 2013); which had the high risk of bias, showed a favorable effect in reducing depressive symptoms (Supplemental eFigure 2). The pooled SMD was −0.32 (95% Cl, −0.64 to −0.01, p = 0.045). There was statistical heterogeneity among the studies (Q = 47.2, p < 0.0001) and the proportion of true heterogeneity was considerable (I^2^ = 81%). For the *meta*-analysis of 10 studies, Egger’s regression test indicated no publication bias (p = 0.24). However, a visual inspection of the funnel plot indicated that one study (Huang et al., 2016) significantly differed from the others (Supplemental eFigure 3). Because of the visually asymmetric funnel plot and heterogeneity of the *meta*-analysis of 10 studies, we conducted a new *meta*-analysis without the RCT (Huang et al., 2016) that was the main reason for the heterogeneity. In the *meta*-analysis of nine studies, the statistically significant effect decreased slightly, but was more accurate (SMD = -0.19, 95% CI = -0.34 to −0.05, p = 0.008) (Fig. 2). The statistical heterogeneity (Q = 16.6, p = 0.03) and the proportion of true heterogeneity (I^2^ = 52%) decreased from high to moderate. The funnel plot (Supplemental eFigure 4) was symmetric and Egger’s regression test indicated no publication bias (p = 0.88). Due to the lower inconsistency and risk of publication bias, we decided to present the *meta*-analysis of nine studies as the main finding.

#### 3.3.2 Subgroup *meta*-analysis according to depression status

This subgroup *meta*-analysis of nine original studies proposed no difference in the effect of intervention between “no depression requirement” populations and populations with elevated depressive symptoms (p = 0.22, see Supplemental eFigure 5 for the forest plot). Among “no depression requirement” populations (k = 7), the pooled SMD was −0.12 (95% Cl, −0.23 to −0.01) with I^2^ of 0%, and among populations with elevated depressive symptoms (k = 2), it was −0.40 (95% Cl, −0.83 to 0.03) with I^2^ of 84%.

### 3.4 The certainty of evidence

We assessed GRADE for the *meta*-analyses of nine studies as low (Table 2). We observed no serious risk of bias, inconsistency, or publication bias. Nevertheless, there were serious differences in the populations and interventions across the studies, which caused indirectness and lowered the general applicability of the results. Correspondingly, there was imprecision in the result, which lowered its clinical relevance: in seven out of the nine studies, the CIs for the mean effect size crossed the line of no effect, and the pooled effect size as such was small.

**Table 2:** Summary of the main findings of the study.

Motivational interviewing-based, health behavior-oriented self-care promotion versus control groups					
P: adults with type 2 diabetes I: motivational interviewing-based health behavior-oriented self-care promotion C: treatment as usual (k = 8) and attention control (k = 1) O: depressive symptoms					
	Change from the baseline in intervention groups*	Change from the baseline in control groups*	Effect size (95% CI)	Participants (studies)	Quality of the evidence (GRADE)

Depressive symptoms	Decreased 23.9%	Decreased 11.9%	SMD −0.19 (-0.34 to −0.05)	1798 (9)	+ + - - LOW ^1, 2^
1 Due to serious indirectness across populations, interventions, and comparators 2 Due to serious imprecision of effect size and inappreciable clinical implication					

*The mean unweighted relative % change in depressive symptoms from the baseline was calculated as follows: ∑n_i_[(s-f)/s]x100/n_i_.

s = depression scores at baseline; f = depression scores at follow up.

## 4 Discussion

We aimed to assess the effects of MI-based, health behavior-oriented self-care promotion on depressive symptoms in adults with T2D. Our findings, based on 11 RCTs and 2,682 participants, suggested that MI-based self-care promotion has a favorable effect on depressive symptoms in the target population. We consider the effect to be preventive since most of the studies did not require clinical depression at the study entry. The main *meta*-analysis was constructed on nine studies, since two studies were excluded to decrease inconsistency and risk of bias. In these nine studies, the interventionist was not a therapist but a person well-trained in MI. The pooled effect size was small but statistically significant, and no considerable heterogeneity or publication bias occurred. However, we assessed the certainty of evidence as low.

To interpret the clinical relevance of our findings, we estimated how much MI-based self-care promotion would have reduced depressive symptoms in the frequently used Beck Depression Inventory (BDI-II) scale, in which scores from 14 to 19 indicate mild depression. The mean unweighted % change in depressive symptoms was –23.9% in the intervention groups and −11.9% in the control groups. On the BDI-II scale, using the cut-off score of 14, these changes would correspond to a reduction of 3.3 points in the intervention group and a reduction of 1.7 points in the control group. From a clinical point of view, these reductions seem considerably minor when considering treatment of depression. When considering only effect sizes, psychotherapies, for instance, are commonly known as an effective treatment for depression compared with TAU though the effect size is small (SMD = -0.31) (Munder et al., 2019). However, the effect size in our main *meta*-analysis was clearly smaller. In the literature, a 20% reduction in depressive symptoms in the BDI-II scale is estimated to be the minimum clinically important change when depressive symptoms are from moderate to higher severity (Kounali et al., 2022). From this perspective, we might consider the effect of MI-based self-care promotion as clinically significant. Furthermore, in most of the original studies, baseline depressive symptoms were low. This might have possibly led to a floor effect, which means that there are not so many scores to be reduced. Only two original studies included elevated depressive symptoms in their inclusion criteria and the effect size tended to be larger in this subgroup. Previously, it has also been proposed that the effect of multicomponent lifestyle interventions might be larger in depressed populations (Wong et al., 2021).

The psychological burden of diabetes diagnosis, stress related to self-care, and possible complications of diabetes increase the risk of depressive symptoms (Nouwen et al., 2011). In our sample, one original study in a population with elevated depressive symptoms applied stress management strategies as a part of the intervention (Huang et al., 2016). This study was, however, excluded from the *meta*-analysis because it appeared to be inconsistent with the others. In the comparison with other studies, its effect size was remarkably large (SMD = -1.95). There may be several reasons for this extremely large impact. First, the study intervention applied not only four sessions of motivational enhancement therapy but also eight additional cognitive behavioral therapy sessions. Second, the intervention included a stress management component. Third, the interventionist was a psychotherapist with probably more advanced skills in psychosocial behavior counseling methods. Fourth, the population had elevated depressive symptoms, which enables a larger effect.

We can also discuss the additional benefit of MI-based self-care promotion in comparison with TAU. Except for one study, the study intervention was more like supportive treatment for TAU. Similarly, most of the studies had TAU as a control group. In our search strategy, we made no restrictions concerning comparison groups. Our results showed that TAU is often applied as a control treatment in studies of this kind. From an ethical point of view, this is understandable when considering the treatment of chronic diseases. The effect size would have probably been larger if the comparison group was a non-active control or wait-list. However, TAU as a comparison realistically describes the additional benefit of MI-based self-care promotion, since the standard treatment for T2D already includes promotion of healthy behaviors. If the small additional benefit provided by MI-based self-care promotion is thought to be preventive, it can be considered clinically relevant.

As a result, what are the elements of MI-based self-care promotion that differ from TAU in T2D? It is impossible to draw precise conclusions because interventions were heterogeneous in their contents: their components, delivery, frequency, and duration varied. Nevertheless, one particular treatment model is not likely to meet everyone's needs.

It is previously reported that personalized care planning has a small positive effect on depressive symptoms among adults with long-term health conditions such as diabetes, renal disease, or depression (Coulter et al., 2015). Similarly, the National Standards for Self-management (Beck et al., 2017) highlight the importance of patient-centeredness and shared-decision making. This means that components in self-care education, delivery, and timing, for example, should be adapted to the individual’s needs. Adequate guidance and individual plans should be applied but the schedule should not be too heavy (May et al., 2014). In addition, to perform and maintain self-care, individuals need sufficient health literacy. This might be supported by offering a variety of information sources (Beck et al., 2017, Young-Hyman et al., 2016). Nonetheless, it seems that a long-lasting effect on health behaviors is not easy to accomplish (Wong et al., 2021, Cezaretto et al., 2016). The difficulty of maintaining behavioral change with less support might be one reason for that. Furthermore, the effect of interventions on self-care behaviors diminishes over time. Thus, booster sessions at different stages of the treatment are needed (Powers et al., 2016). In our review, all interventions were patient-oriented; included a baseline assessment of health behaviors; an exchange of information between an interventionist and patient; individualized goal setting; educational materials; follow-ups; and feedback. These types of elements are included, for example, in the 5A method of supporting smoking cessation (Fiore et al., 2008), but also recommended in self-management standards (Beck et al., 2017).

Patient-centeredness, a non-authoritarian approach, and supporting of motivation and self-efficacy are essential elements of self-care promotion, but also the basic principles in MI. Furthermore, self-efficacy is known to be associated with better performance in self-care related health behaviors such as diet and exercise (King et al., 2010). In fact, self-efficacy has been recommended to regularly be evaluated and supported to achieve successful self-care (Young-Hyman et al., 2016). Thus, it is likely that the MI approach plays a key role in explaining the impact of MI-based self-care promotion on depressive symptoms. Previously, it has been proposed that including a psychological element in T2D self-care education is one of the key elements in preventing depressive symptoms (Guérin et al., 2019) and providing psychosocial care (Young-Hyman et al., 2016). Depression as such causes lack of motivation, which is why actions to enhance motivation are important.

The interventionists in the studies included in the *meta*-analysis were not therapists but persons well-trained in MI. In primary health care, it seems to be worth training people working with T2D patients to conduct MI. Self-care guidance already takes place in primary care settings, but it might be intensified by including MI as a part of the usual treatment. To only give instructions is not enough; rather patients must be empowered to better take care of themselves. We suggest that by means of MI, professionals other than therapists might gain the basic skills needed to possibly prevent depressive symptoms in the T2D population. The preventive perspective is not far-fetched, as healthy behaviors have been found to increase mental well-being (Velten et al., 2018) and prevent depressive symptoms (Gómez-Gómez et al., 2020).

### 4.1 Strengths and limitations

The methodology of our systematic review and *meta*-analysis was transparent. Furthermore, GRADE was assessed, which facilitates the applicability of the main findings in the treatment of patients. We also examined the sources of heterogeneity. The search strategy was comprehensive and resulted in no publication bias. The populations of the original studies were suitable for answering the research question. Consequently, the main findings of our study can be considered a valid and reliable synthesis of the original studies.

We acknowledge some limitations. First, despite the good overall quality of the original studies, there was some indirectness and imprecision in the results, which limited the certainty of the conclusions. Second, the diversity of interventions and populations resulted in true heterogeneity across the studies. Third, only a few studies had examined the effects of self-care promotion in clinically depressed populations, which is why our study did not manage to build knowledge with respect to this highly important group. However, this is also a strength since our study now strengthened the evidence that there is a lack of studies in depressed populations. Fourth, in preliminary *meta*-regressions, the depression status explained the effect of lifestyle interventions, but the statistical test for subgroup differences resulted in a p-value of 0.22, which complicated the interpretation. One possible explanation might be that there were depressed individuals also in the studies that did not require depressive symptoms as an inclusion criterion. Fifth, the content of TAU varied across the original studies, probably following different diabetes treatment guidelines, which to some extent questioned the role of TAU as a comparator. Sixth, our decision to include only studies that reported the use of MI may have overlooked some relevant studies with varying motivational components. However, there are many sources of heterogeneity among complex interventions (e.g., setting, types of participants, implementation, framework). We applied this inclusion criterion for reducing the variation of interventions. In addition, the outcome was included in the search strategy (Frandsen et al., 2020), and we did not search for gray literature. These restrictions may have led to excluding some information that would have enhanced our observations.

### 4.2 Future research

Future trials should examine the effects of MI-based health behavior-oriented self-care promotion on depressive symptoms in clinically depressed populations. As stress management may be an important component in reducing and preventing depressive symptoms in the T2D population, this should be studied further. In addition, to examine the preventive effect of MI-based self-care promotion on depressive symptoms, studies with longer follow-up times would be encouraged.

## 5 Conclusion

The present systematic review and *meta*-analysis suggest that MI-based self-care promotion has a statistically significant effect on depressive symptoms among adults with T2D. The effect might be considered to be preventive since most of the studies in the review did not require clinical depression at the study entry. MI-based self-care promotion appears to offer a potential method to increase the effectiveness of standard health promotion in preventing depressive symptoms and it is applicable for persons with varying backgrounds. However, the certainty of evidence was assessed as low. In future trials, the effect of MI-based self-care interventions on depression should be studied in clinically depressive populations.

## Funding

This research did not receive any specific grant from funding agencies in the public, commercial, or not-for-profit sectors.

## CRediT authorship contribution statement

**Ulla Mikkonen:** Conceptualization, Methodology, Formal analysis, Investigation, Writing – original draft, Writing – review & editing. **Ari Voutilainen:** Conceptualization, Methodology, Formal analysis, Investigation, Supervision, Visualization, Writing – review & editing. **Tuomas Mikola:** Formal analysis, Investigation, Writing – review & editing. **Johanna Roponen:** Formal analysis, Investigation, Writing – review & editing. **Sanna Rajapolvi:** Conceptualization, Formal analysis, Investigation. **Soili M. Lehto:** Conceptualization, Investigation, Methodology, Supervision, Writing – review & editing. **Anu Ruusunen:** Conceptualization, Formal analysis, Investigation, Methodology, Supervision, Writing – review & editing. **Pekka Mäntyselkä:** Conceptualization, Investigation, Supervision, Writing – review & editing.

## Declaration of Competing Interest

The authors declare that they have no known competing financial interests or personal relationships that could have appeared to influence the work reported in this paper.

## Data Availability

Data will be made available on request.

## References

[b0005] Ali M.K., Chwastiak L., Poongothai S., Emmert-Fees K.M.F., Patel S.A., Anjana R.M., Sagar R., Shankar R., Sridhar G.R., Kosuri M., Sosale A.R., Sosale B., Rao D., Tandon N., Narayan K.M.V., Mohan V. (2020). Effect of a collaborative care model on depressive symptoms and glycated hemoglobin, blood pressure, and serum cholesterol among patients with depression and diabetes in India: the independent randomized clinical trial. J. Am. Med. Assoc..

[b0010] Anderson R.J., Freedland K.E., Clouse R.E., Lustman P.J. (2001). The prevalence of comorbid depression in adults with diabetes: a meta-analysis. Diabetes Care.

[b0015] Azami G., Soh K.L., Sazlina S.G., Salmiah M.S., Aazami S., Mozafari M., Taghinejad H. (2018). Effect of a nurse-led diabetes self-management education program on glycosylated hemoglobin among adults with type 2 diabetes. J. Diabetes Res..

[b0020] Balshem H., Helfand M., Schünemann H.J., Oxman A.D., Kunz R., Brozek J., Vist G.E., Falck-Ytter Y., Meerpohl J., Norris S. (2011). GRADE guidelines: 3. Rating the quality of evidence. J. Clin. Epidemiol..

[b0025] Bandura A. (1997).

[b0030] Beck J., Greenwood D.A., Blanton L., Bollinger S.T., Butcher M.K., Condon J.E., Cypress M., Faulkner P., Fischl A.H., Francis T., Kolb L.E., Lavin-Tompkins J.M., MacLeod J., Maryniuk M., Mensing C., Orzeck E.A., Pope D.D., Pulizzi J.L., Reed A.A., Rhinehart A.S., Siminerio L., Wang J. (2017). 2017 national standards for diabetes self-management education and support. Diabetes Care.

[b0035] Cezaretto A., Ferreira S.R.G., Sharma S., Sadeghirad B., Kolahdooz F. (2016). Impact of lifestyle interventions on depressive symptoms in individuals at-risk of, or with, type 2 diabetes mellitus: a systematic review and meta-analysis of randomized controlled trials. Nutr. Metab. Cardiovasc. Dis..

[b0040] Coulter A, Entwistle VA, Eccles A, Ryan S, Shepperd S, Perera R. Personalised care planning for adults with chronic or long-term health conditions. Cochrane Database Syst Rev 2015:CD010523. https://doi.org/10.1002/14651858.CD010523.pub2. 10.1002/14651858.CD010523.pub2 PMC6486144 25733495

[b0045] Covidence systematic review software. Veritas Health Innovation, Melbourne, Australia. Available at https://www.covidence.org. Accessibility verified August 7, 2023.

[b0050] Döbler A., Herbeck Belnap B., Pollmann H., Farin E., Raspe H., Mittag O. (2018). Telephone-delivered lifestyle support with action planning and motivational interviewing techniques to improve rehabilitation outcomes. Rehabil. Psychol..

[b0055] Egede L.E., Zheng D., Simpson K. (2002). Comorbid depression is associated with increased health care use and expenditures in individuals with diabetes. Diabetes Care.

[b0060] Egger M., Davey Smith G., Schneider M., Minder C. (1997). Bias in meta-analysis detected by a simple, graphical test. BMJ.

[b0065] Fiore MC, Jaén CR, Baker TB, et al. Treating Tobacco Use and Dependence: 2008 Update. Clinical Practice Guideline. Rockville, MD: U.S. Department of Health and Human Services. Public Health Service. May 2008.

[b0070] Firth J., Marx W., Dash S., Carney R., Teasdale S.B., Solmi M., Stubbs B., Schuch F.B., Carvalho A.F., Jacka F., Sarris J. (2019). The effects of dietary improvement on symptoms of depression and anxiety: a meta-analysis of randomized controlled trials. Psychosom. Med..

[b0075] Frandsen T.F., Bruun Nielsen M.F., Lindhardt C.L., Eriksen M.B. (2020). Using the full PICO model as a search tool for systematic reviews resulted in lower recall for some PICO elements. J. Clin. Epidemiol..

[b0080] Gabbay R.A., Añel-Tiangco R.M., Dellasega C., Mauger D.T., Adelman A., Van Horn D.H.A. (2013). Diabetes nurse case management and motivational interviewing for change (DYNAMIC): results of a 2-year randomized controlled pragmatic trial. J. Diabetes.

[b0085] Glasgow R.E., Nutting P.A., Toobert D.J., King D.K., Strycker L.A., Jex M., O'Neill C., Whitesides H., Merenich J. (2006). Effects of a brief computer-assisted diabetes self-management intervention on dietary, biological and quality-of-life outcomes. Chronic. Illn..

[b0090] Golden S.H., Lazo M., Carnethon M., Bertoni A.G., Schreiner P.J., Diez Roux A.V. (2008). Examining a bidirectional association between depressive symptoms and diabetes. J. Am. Med. Assoc..

[b0095] Gómez-Gómez I., Bellón J.Á., Resurrección D.M., Cuijpers P., Moreno-Peral P., Rigabert A., Maderuelo-Fernández J.Á., Motrico E. (2020). Effectiveness of universal multiple-risk lifestyle interventions in reducing depressive symptoms: Systematic review and meta-analysis. Prev. Med..

[b0100] Gonzalez J.S., Peyrot M., McCarl L.A., Collins E.M., Serpa L., Mimiaga M.J. (2008). Depression and diabetes treatment nonadherence: a meta-analysis. Diabetes Care.

[b0105] Guérin E., Jaafar H., Amrani L., Prud'homme D., Aguer C. (2019;7:35.). Intervention strategies for prevention of comorbid depression among individuals with type 2 diabetes: a scoping review. Front Public Health.

[b0110] Haddaway N.R., Page M.J., Pritchard C.C., McGuinness L.A. (2022). PRISMA2020: an R package and Shiny app for producing PRISMA 2020-compliant flow diagrams, with interactivity for optimised digital transparency and Open Synthesis. Campbell Syst. Rev..

[b0115] Higgins JPT, Thomas J, Chandler J, et al. Cochrane Handbook for Systematic Reviews of Interventions version 6.3 (updated February 2022). Cochrane, 2022. Available from https://www.training.cochrane.org/handbook. Accessibility verified August 7, 2023.

[b0120] Himelhoch S., Weller W.E., Wu A.W., Anderson G.F., Cooper L.A. (2004). Chronic medical illness, depression, and use of acute medical services among Medicare beneficiaries. Med. Care.

[b0125] Holmen H, Torbjørnsen A, Wahl AK, Jenum AK, Småstuen MC, Arsand E, et al. A Mobile Health Intervention for Self-Management and Lifestyle Change for Persons With Type 2 Diabetes, Part 2: One-Year Results From the Norwegian Randomized Controlled Trial RENEWING HEALTH. JMIR Mhealth Uhealth 2014;2:e57. https://doi.org/10.2196/mhealth.3882. 10.2196/mhealth.3882 PMC4275495 25499872

[b0130] Huang C.-Y., Lai H.-L., Chen C.-I., Lu Y.-C., Li S.-C., Wang L.-W., Su Y.i. (2016). Effects of motivational enhancement therapy plus cognitive behaviour therapy on depressive symptoms and health-related quality of life in adults with type II diabetes mellitus: a randomised controlled trial. Qual. Life Res..

[b0135] Jacka F.N., Berk M. (2013). Depression, diet and exercise. Med. J. Aust..

[b0140] Katon W.J., Lin E.H.B., Von Korff M., Ciechanowski P., Ludman E.J., Young B., Peterson D.o., Rutter C.M., McGregor M., McCulloch D. (2010). Collaborative care for patients with depression and chronic illnesses. N. Engl. J. Med..

[b0145] King D.K., Glasgow R.E., Toobert D.J., Strycker L.A., Estabrooks P.A., Osuna D. (2010). Self-efficacy, problem solving, and social-environmental support are associated with diabetes self-management behaviors. Diabetes Care.

[b0150] Knol M.J., Heerdink E.R., Egberts A.C.G., Geerlings M.I., Gorter K.J., Numans M.E., Grobbee D.E., Klungel O.H., Burger H. (2007). Depressive symptoms in subjects with diagnosed and undiagnosed type 2 diabetes. Psychosom. Med..

[b0155] Kounali D., Button K.S., Lewis G., Gilbody S., Kessler D., Araya R., Duffy L., Lanham P., Peters T.J., Wiles N., Lewis G. (2022). How much change is enough? Evidence from a longitudinal study on depression in UK primary care. Psychol. Med..

[b0160] Kvam S., Kleppe C.L., Nordhus I.H., Hovland A. (2016). Exercise as a treatment for depression: a meta-analysis. J. Affect. Disord..

[b0165] May C.R., Eton D.T., Boehmer K., Gallacher K., Hunt K., MacDonald S., Mair F.S., May C.M., Montori V.M., Richardson A., Rogers A.E., Shippee N. (2014). Rethinking the patient: using Burden of Treatment Theory to understand the changing dynamics of illness. BMC Health Serv. Res..

[b0170] Mezuk B., Eaton W.W., Albrecht S., Golden S.H. (2008). Depression and type 2 diabetes over the lifespan: a meta-analysis. Diabetes Care.

[b0175] Miller W.R. (1983). Motivational interviewing with problem drinkers. Behav. Cogn. Psychother..

[b0180] Miller W.R., Rollnick S. (1991).

[b0185] Morrison A., Polisena J., Husereau D., Moulton K., Clark M., Fiander M., Mierzwinski-Urban M., Clifford T., Hutton B., Rabb D. (2012). The effect of English-language restriction on systematic review-based meta-analyses: a systematic review of empirical studies. Int. J. Technol. Assess. Health Care.

[b0190] Munder T., Flückiger C., Leichsenring F., Abbass A.A., Hilsenroth M.J., Luyten P., Rabung S., Steinert C., Wampold B.E. (2019). Is psychotherapy effective? A re-analysis of treatments for depression. Epidemiol. Psychiatr. Sci..

[b0195] Nguyen M.M., Perlman G., Kim N., Wu C.-Y., Daher V., Zhou A., Mathers E.H., Anita N.Z., Lanctôt K.L., Herrmann N., Pakosh M., Swardfager W. (2021). Depression in type 2 diabetes: a systematic review and meta-analysis of blood inflammatory markers. Psychoneuroendocrinology.

[b0200] Nouwen A., Nefs G., Caramlau I., Connock M., Winkley K., Lloyd C.E., Peyrot M., Pouwer F. (2011). Prevalence of depression in individuals with impaired glucose metabolism or undiagnosed diabetes: a systematic review and meta-analysis of the European Depression in Diabetes (EDID) Research Consortium. Diabetes Care.

[b0205] Nouwen A., Adriaanse M.C., van Dam K., Iversen M.M., Viechtbauer W., Peyrot M., Caramlau I., Kokoszka A., Kanc K., de Groot M., Nefs G., Pouwer F. (2019). Longitudinal associations between depression and diabetes complications: a systematic review and meta-analysis. Diabet. Med..

[b0210] Otsu H., Moriyama M. (2011). Effectiveness of an educational self-management program for outpatients with chronic heart failure. Jpn. J. Nurs. Sci..

[b0215] Page MJ, McKenzie JE, Bossuyt PM, Boutron I, Hoffmann TC, Mulrow CD, et al. The PRISMA 2020 statement: an updated guideline for reporting systematic reviews. BMJ 2021;372:n71. https://doi.org/10.1136/bmj.n71. 10.1136/bmj.n71 PMC8005924 33782057

[b0220] Park M., Katon W.J., Wolf F.M. (2013). Depression and risk of mortality in individuals with diabetes: a meta-analysis and systematic review. Gen. Hosp. Psychiatry.

[b0225] Peters M., Potter C.M., Kelly L., Fitzpatrick R. (2019). Self-efficacy and health-related quality of life: a cross-sectional study of primary care patients with multi-morbidity. Health Qual. Life Outcomes.

[b0230] Powers M.A., Bardsley J., Cypress M., Duker P., Funnell M.M., Fischl A.H., Maryniuk M.D., Siminerio L., Vivian E. (2016). Diabetes self-management education and support in type 2 diabetes: a joint position statement of the american diabetes association, the american association of diabetes educators, and the academy of nutrition and dietetics. Clin Diabetes.

[b0235] R Core Team (2020).

[b0240] Riper H., Andersson G., Hunter S.B., de Wit J., Berking M., Cuijpers P. (2014). Treatment of comorbid alcohol use disorders and depression with cognitive-behavioural therapy and motivational interviewing: a meta-analysis. Addiction.

[b0245] Rotella F., Mannucci E. (2013). Depression as a risk factor for diabetes: a meta-analysis of longitudinal studies. J. Clin. Psychiatry.

[b0250] Sackett D.L. (1989). Rules of evidence and clinical recommendations on the use of antithrombotic agents. Chest.

[b0255] Sarris J., O’Neil A., Coulson C.E., Schweitzer I., Berk M. (2014). Lifestyle medicine for depression. BMC Psychiatry.

[b0260] Sarris J., Thomson R., Hargraves F., Eaton M., de Manincor M., Veronese N., Solmi M., Stubbs B., Yung A.R., Firth J. (2020). Multiple lifestyle factors and depressed mood: a cross-sectional and longitudinal analysis of the UK Biobank (N = 84,860). BMC Med..

[b0265] Schwalbe C.S., Oh H.Y., Zweben A. (2014). Sustaining motivational interviewing: a meta-analysis of training studies. Addiction.

[b0270] Schwarzer G., Carpenter J.R., Rücker G. (2015).

[b0275] Sterne JAC, Savović J, Page MJ, Elbers RG, Blencowe NS, Boutron I, et al. RoB 2: a revised tool for assessing risk of bias in randomised trials. BMJ 2019;366:l4898. https://doi.org/10.1136/bmj.l4898. 10.1136/bmj.l4898 31462531

[b0280] Swoboda C.M., Miller C.K., Wills C.E. (2017). Impact of a goal setting and decision support telephone coaching intervention on diet, psychosocial, and decision outcomes among people with type 2 diabetes. Patient Educ. Couns..

[b0285] Tusa N., Koponen H., Kautiainen H., Korniloff K., Raatikainen I., Elfving P., Vanhala M., Mäntyselkä P. (2019). The profiles of health care utilization among a non-depressed population and patients with depressive symptoms with and without clinical depression. Scand. J. Prim. Health Care.

[b0290] van Agtmaal M.J.M., Houben A.J.H.M., Pouwer F., Stehouwer C.D.A., Schram M.T. (2017). Association of microvascular dysfunction with late-life depression: a systematic review and meta-analysis. JAMA Psychiat..

[b0295] van der Wulp I., de Leeuw J.R.J., Gorter K.J., Rutten G.E.H.M. (2012). Effectiveness of peer-led self-management coaching for patients recently diagnosed with Type 2 diabetes mellitus in primary care: a randomized controlled trial. Diabet. Med..

[b0300] Velten J., Bieda A., Scholten S., Wannemüller A., Margraf J. (2018). Lifestyle choices and mental health: a longitudinal survey with German and Chinese students. BMC Public Health.

[b0305] Wang C., Lang J., Xuan L., Li X., Zhang L. (2017). The effect of health literacy and self-management efficacy on the health-related quality of life of hypertensive patients in a western rural area of China: a cross-sectional study. Int. J. Equity Health.

[b0310] Wang J., Zhou D., Dai Z., Li X. (2021). Association between systemic immune-inflammation index and diabetic depression. Clin. Interv. Aging.

[b0315] Wong V.-W.-H., Ho F.-Y.-Y., Shi N.-K., Sarris J., Chung K.-F., Yeung W.-F. (2021). Lifestyle medicine for depression: A meta-analysis of randomized controlled trials. J. Affect. Disord..

[b0320] Young H.M., Miyamoto S., Dharmar M., Tang-Feldman Y. (2020). Nurse coaching and mobile health compared with usual care to improve diabetes self-efficacy for persons with type 2 diabetes: randomized controlled trial. JMIR Mhealth Uhealth.

[b0325] Young-Hyman D., de Groot M., Hill-Briggs F., Gonzalez J.S., Hood K., Peyrot M. (2016). Psychosocial care for people with diabetes: a position statement of the american diabetes association. Diabetes Care.

