# Supplementary material for: The effects of motivational self-care promotion on depressive symptoms among adults with type 2 diabetes: A systematic review and meta-analysis
Source: Prev Med Rep. 2023 Sep 20;36:102431. doi: 10.1016/j.pmedr.2023.102431 (PMC10523005; doi:10.1016/j.pmedr.2023.102431)
Supplement: Supplementary data 1 [file mmc1.docx]

Supplemental online only material

The effects of motivational self-care promotion on depressive symptoms among adults with type 2 diabetes: A systematic review and meta-analysis.

Content:

eTable 1. PRISMA 2020 Checklist

eTable 2. Full search strategies for all databases

eTable 3. Excluded eligible studies

eTable 4. The details of interventions and funding

eFigure 1. Risk of bias across studies

eFigure 2. Forest plot for 10 studies combined

eFigure 3. Funnel plot for10 studies combined

eFigure 4. Funnel plot for 9 studies combined

eFigure 5. Forest plot for subgroup meta-analysis of 9 studies combined

**Supplemental eTable 1.** PRISMA 2020 Checklist (1).

| **Section and Topic** | **Item #** | **Checklist item** | **Location where item is reported** |
| --- | --- | --- | --- |
| **TITLE** | | |  |
| Title | 1 | Identify the report as a systematic review. | 1 |
| **ABSTRACT** | | |  |
| Abstract | 2 | See the PRISMA 2020 for Abstracts checklist. | 2 |
| **INTRODUCTION** | | |  |
| Rationale | 3 | Describe the rationale for the review in the context of existing knowledge. | 3-4 |
| Objectives | 4 | Provide an explicit statement of the objective(s) or question(s) the review addresses. | 4 |
| **METHODS** | | |  |
| Eligibility criteria | 5 | Specify the inclusion and exclusion criteria for the review and how studies were grouped for the syntheses. | 5-6 |
| Information sources | 6 | Specify all databases, registers, websites, organisations, reference lists and other sources searched or consulted to identify studies. Specify the date when each source was last searched or consulted. | 5 |
| Search strategy | 7 | Present the full search strategies for all databases, registers and websites, including any filters and limits used. | eTable 2 |
| Selection process | 8 | Specify the methods used to decide whether a study met the inclusion criteria of the review, including how many reviewers screened each record and each report retrieved, whether they worked independently, and if applicable, details of automation tools used in the process. | 5-6 |
| Data collection process | 9 | Specify the methods used to collect data from reports, including how many reviewers collected data from each report, whether they worked independently, any processes for obtaining or confirming data from study investigators, and if applicable, details of automation tools used in the process. | 5-6 |
| Data items | 10a | List and define all outcomes for which data were sought. Specify whether all results that were compatible with each outcome domain in each study were sought (e.g. for all measures, time points, analyses), and if not, the methods used to decide which results to collect. | 6 |
|  | 10b | List and define all other variables for which data were sought (e.g. participant and intervention characteristics, funding sources). Describe any assumptions made about any missing or unclear information. | 6 |
| Study risk of bias assessment | 11 | Specify the methods used to assess risk of bias in the included studies, including details of the tool(s) used, how many reviewers assessed each study and whether they worked independently, and if applicable, details of automation tools used in the process. | 6 |
| Effect measures | 12 | Specify for each outcome the effect measure(s) (e.g. risk ratio, mean difference) used in the synthesis or presentation of results. | 6-7 |
| Synthesis methods | 13a | Describe the processes used to decide which studies were eligible for each synthesis (e.g. tabulating the study intervention characteristics and comparing against the planned groups for each synthesis (item #5)). | 6-7 |
|  | 13b | Describe any methods required to prepare the data for presentation or synthesis, such as handling of missing summary statistics, or data conversions. | 6-7 |
|  | 13c | Describe any methods used to tabulate or visually display results of individual studies and syntheses. | 6-7 |
|  | 13d | Describe any methods used to synthesize results and provide a rationale for the choice(s). If meta-analysis was performed, describe the model(s), method(s) to identify the presence and extent of statistical heterogeneity, and software package(s) used. | 6-7 |
|  | 13e | Describe any methods used to explore possible causes of heterogeneity among study results (e.g. subgroup analysis, meta-regression). | 6-7 |
|  | 13f | Describe any sensitivity analyses conducted to assess robustness of the synthesized results. | 6-7 |
| Reporting bias assessment | 14 | Describe any methods used to assess risk of bias due to missing results in a synthesis (arising from reporting biases). | 6-7, 9 |
| Certainty assessment | 15 | Describe any methods used to assess certainty (or confidence) in the body of evidence for an outcome.6, | 6, 10 |
| **RESULTS** | | |  |
| Study selection | 16a | Describe the results of the search and selection process, from the number of records identified in the search to the number of studies included in the review, ideally using a flow diagram. | 8-9, Fig 1 |
|  | 16b | Cite studies that might appear to meet the inclusion criteria, but which were excluded, and explain why they were excluded. | 7-8, eTable 3 |
| Study characteristics | 17 | Cite each included study and present its characteristics. | 8-9, Table 1, eTable 4 |
| Risk of bias in studies | 18 | Present assessments of risk of bias for each included study. | 9, eFig 1 |
| Results of individual studies | 19 | For all outcomes, present, for each study: (a) summary statistics for each group (where appropriate) and (b) an effect estimate and its precision (e.g. confidence/credible interval), ideally using structured tables or plots. | 9-10, Fig 2, eFig 2-5 |
| Results of syntheses | 20a | For each synthesis, briefly summarise the characteristics and risk of bias among contributing studies. | 9-10 |
|  | 20b | Present results of all statistical syntheses conducted. If meta-analysis was done, present for each the summary estimate and its precision (e.g. confidence/credible interval) and measures of statistical heterogeneity. If comparing groups, describe the direction of the effect. | 9-10, Fig 2, eFig2-5 |
|  | 20c | Present results of all investigations of possible causes of heterogeneity among study results. | 8-10 |
|  | 20d | Present results of all sensitivity analyses conducted to assess the robustness of the synthesized results. | N/A |
| Reporting biases | 21 | Present assessments of risk of bias due to missing results (arising from reporting biases) for each synthesis assessed. | N/A |
| Certainty of evidence | 22 | Present assessments of certainty (or confidence) in the body of evidence for each outcome assessed. | 10, Table 2 |
| **DISCUSSION** | | |  |
| Discussion | 23a | Provide a general interpretation of the results in the context of other evidence. | 10-13 |
|  | 23b | Discuss any limitations of the evidence included in the review. | 13-14 |
|  | 23c | Discuss any limitations of the review processes used. | 13-14 |
|  | 23d | Discuss implications of the results for practice, policy, and future research. | 10-14 |
| **OTHER INFORMATION** | | |  |
| Registration and protocol | 24a | Provide registration information for the review, including register name and registration number, or state that the review was not registered. | 5 |
|  | 24b | Indicate where the review protocol can be accessed, or state that a protocol was not prepared. | 5 |
|  | 24c | Describe and explain any amendments to information provided at registration or in the protocol. | 5 (protocol) |
| Support | 25 | Describe sources of financial or non-financial support for the review, and the role of the funders or sponsors in the review. | 15 |
| Competing interests | 26 | Declare any competing interests of review authors. | 15 |
| Availability of data, code and other materials | 27 | Report which of the following are publicly available and where they can be found: template data collection forms; data extracted from included studies; data used for all analyses; analytic code; any other materials used in the review. | Available from the corresponding author |

*From:*  Page MJ, McKenzie JE, Bossuyt PM, et al. The PRISMA 2020 statement: an updated guideline for reporting systematic reviews. *BMJ*. 2021;372:n71.

**Supplemental eTable 2**. Full search strategies for all databases. Primary search (in April 2021) and re-search (in February 2023).

| Pubmed (MEDLINE) |
| --- |
| #1 Depression[mh] OR depress*[tw]  #2 intervention*[tw] OR therap*[tw] OR treatment*[tw]  #3 "Life Style"[mh] OR "life style*"[tw] OR lifestyle*[tw]  #4 Exercise[mh] OR Sports[mh] OR exercise*[tw] OR sport*[tw] OR "physical activit*"[tw] OR fitness[tw] OR walking[tw] OR jogging[tw] OR jogger*[tw] OR running[tw] OR runner*[tw] OR gym[tw] OR gyms[tw] OR gymnastic*[tw] OR swim*[tw] OR skiing[tw] OR skier*[tw] OR "weight lift*"[tw] OR skating[tw] OR hockey[tw] OR football[tw] OR soccer[tw] OR basketball[tw] OR volleyball[tw]  #5 "Diet, Food, and Nutrition"[mh] OR diet*[tw] OR food*[tw] OR nutrit*[tw] OR nutrient*[tw] OR eating[tw] OR meal*[tw] OR breakfast*[tw] OR brunch*[tw] OR lunch*[tw] OR dinner*[tw] OR supper*[tw]  #6 Sleep[mh] OR Dyssomnias[mh] OR Parasomnias[mh] OR sleep*[tw] OR dyssomnia*[tw] OR parasomnia*[tw] OR somnole*[tw]  #7 Smoking[mh] OR Tobacco[mh] OR smok*[tw] OR tobacco[tw] OR cigar*[tw] OR "e-cig*"[tw] OR snuff*[tw] OR "nicotine addict*"[tw]  #8 "Quality of Life"[mh] OR "quality of life"[tw] OR "life quality"[tw] OR "body weight"[tw] OR "body mass index"[tw] OR bmi[tw] OR "waist circumference"[tw] OR "blood pressure"[tw] OR hypertens*[tw] OR "lipids/blood"[mh] OR "blood lipid*"[tw] OR "blood glucose"[tw]  #9 "Stress, Psychological"[mh] OR "Psychological Distress"[mh] OR Anxiety[mh] OR stress*[tw] OR distress*[tw] OR anxiet*[tw]  #10 "Treatment Adherence and Compliance"[mh] OR adherence[tw] OR compliance[tw] OR nonadherence[tw] OR noncompliance[tw] OR acceptance[tw] OR satisfaction[tw] OR refus*[tw] OR negative[tw] OR adverse[tw] OR worsen*[tw]  #11 #2 OR #3 OR #4 OR #5 OR #6 OR #7 OR #8 OR #9 OR #10  #12 "Motivational Interviewing"[mh] OR "motivational interview*"[tw] OR "motivation interview*"[tw]  #13 #1 AND #11 AND #12  #14 #13 AND (Adult[mh] OR adult[tw] OR adults[tw])  #15 #13 NOT (Child[mh] OR Adolescent[mh] OR child*[tw] OR adolescen*[tw] OR youth[tw])  #16 #14 OR #15  #17 "Randomized Controlled Trials as Topic"[mh] OR "Random Allocation"[mh] OR "Randomized Controlled Trial"[pt] OR "Clinical Trials as Topic"[mh] OR "Clinical Trial"[pt] OR "Double-Blind Method"[mh] OR Placebos[mh]  #18 rct[tw] OR rcts[tw] OR "randomized controlled"[tw] OR "randomised controlled"[tw] OR "random control"[tw] OR "randomized trial*"[tw] OR "randomised trial*"[tw] OR "random allocation"[tw] OR randomly[tw] OR randomisation*[tw] OR randomization*[tw] OR "clinical trial*"[tw] OR "controlled trial*"[tw] OR "control trial*"[tw] OR "double blind*"[tw] OR "double mask*"[tw] OR placebo*[tw]  #19 #17 OR #18  #20 #16 AND #19  #21 #20 AND English[la] |
| Scopus |
| #1 TITLE-ABS-KEY(depress*)  #2 TITLE-ABS-KEY(intervention* OR therap* OR treatment*)  #3 TITLE-ABS-KEY("life style*" OR lifestyle*)  #4 TITLE-ABS-KEY(exercise* OR sport* OR "physical activit*" OR fitness OR walking OR jogging OR jogger* OR running OR runner* OR gym OR gyms OR gymnastic* OR swim* OR skiing OR skier* OR "weight lift*" OR skating OR hockey OR football OR soccer OR basketball OR volleyball)  #5 TITLE-ABS-KEY(diet* OR food* OR nutrit* OR nutrient* OR eating OR meal* OR breakfast* OR brunch* OR lunch* OR dinner* OR supper*)  #6 TITLE-ABS-KEY(sleep* OR dyssomnia* OR parasomnia* OR somnole*)  #7 TITLE-ABS-KEY(smok* OR tobacco OR cigar* OR "e-cig*" OR snuff* OR "nicotine addict*")  #8 TITLE-ABS-KEY("quality of life" OR "life quality" OR "body weight" OR "body mass index" OR bmi OR "waist circumference" OR "blood pressure" OR hypertens* OR (lipid* W/2 blood) OR (blood W/2 glucose))  #9 TITLE-ABS-KEY(stress* OR distress* OR anxiet*)  #10 TITLE-ABS-KEY(adherence OR compliance OR nonadherence OR noncompliance OR acceptance OR satisfaction OR refus* OR negative OR adverse OR worsen*)  #11 #2 OR #3 OR #4 OR #5 OR #6 OR #7 OR #8 OR #9 OR #10  #12 TITLE-ABS-KEY("motivational interview*" OR "motivation interview*")  #13 #1 AND #11 AND #12  #14 TITLE-ABS-KEY(adult OR adults)  #15 #13 AND #14  #16 TITLE-ABS-KEY(child* OR adolescen* OR youth)  #17 #13 AND NOT #16  #18 #15 OR #17  #19 TITLE-ABS-KEY(rct OR rcts OR "randomized controlled" OR "randomised controlled" OR "random control" OR "randomized trial*" OR "randomised trial*" OR "random allocation" OR randomly OR randomisation* OR randomization* OR "clinical trial*" OR "controlled trial*" OR "control trial*" OR "double blind*" OR "double mask*" OR placebo*)  #20 #18 AND #19  #21 LANGUAGE(English)  #22 #20 AND #21 |
| PsycINFO (EBSCO) |
| S1 depress*  S2 intervention* OR therap* OR treatment*  S3 "life style*" OR lifestyle*  S4 exercise* OR sport* OR "physical activit*" OR fitness OR walking OR jogging OR jogger* OR running OR runner* OR gym OR gyms OR gymnastic* OR swim* OR skiing OR skier* OR "weight lift*" OR skating OR hockey OR football OR soccer OR basketball OR volleyball  S5 diet* OR food* OR nutrit* OR nutrient* OR eating OR meal* OR breakfast* OR brunch* OR lunch* OR dinner* OR supper*  S6 sleep* OR dyssomnia* OR parasomnia* OR somnole*  S7 smok* OR tobacco OR cigar* OR "e-cig*" OR snuff* OR "nicotine addict*"  S8 "quality of life" OR "life quality" OR "body weight" OR "body mass index" OR bmi OR "waist circumference" OR "blood pressure" OR hypertens* OR (lipid* N2 blood) OR (blood N2 glucose)  S9 stress* OR distress* OR anxiet*  S10 adherence OR compliance OR nonadherence OR noncompliance OR acceptance OR satisfaction OR refus* OR negative OR adverse OR worsen*  S11 S2 OR S3 OR S4 OR S5 OR S6 OR S7 OR S8 OR S9 OR S10  S12 "motivational interview*" OR "motivation interview*"  S13 S1 AND S11 AND S12  S14 adult OR adults  S15 S13 AND S14  S16 child* OR adolescen* OR youth  S17 S13 NOT S16  S18 S15 OR S17  S19 rct OR rcts OR "randomized controlled" OR "randomised controlled" OR "random control" OR "randomized trial*" OR "randomised trial*" OR "random allocation" OR randomly OR randomisation* OR randomization* OR "clinical trial*" OR "controlled trial*" OR "control trial*" OR "double blind*" OR "double mask*" OR placebo*  S20 S18 AND S19  S21 S20 AND limit to English |
| CINAHL (EBSCO) |
| S1 depress*  S2 intervention* OR therap* OR treatment*  S3 "life style*" OR lifestyle*  S4 exercise* OR sport* OR "physical activit*" OR fitness OR walking OR jogging OR jogger* OR running OR runner* OR gym OR gyms OR gymnastic* OR swim* OR skiing OR skier* OR "weight lift*" OR skating OR hockey OR football OR soccer OR basketball OR volleyball  S5 diet* OR food* OR nutrit* OR nutrient* OR eating OR meal* OR breakfast* OR brunch* OR lunch* OR dinner* OR supper*  S6 sleep* OR dyssomnia* OR parasomnia* OR somnole*  S7 smok* OR tobacco OR cigar* OR "e-cig*" OR snuff* OR "nicotine addict*"  S8 "quality of life" OR "life quality" OR "body weight" OR "body mass index" OR bmi OR "waist circumference" OR "blood pressure" OR hypertens* OR (lipid* N2 blood) OR (blood N2 glucose)  S9 stress* OR distress* OR anxiet*  S10 adherence OR compliance OR nonadherence OR noncompliance OR acceptance OR satisfaction OR refus* OR negative OR adverse OR worsen*  S11 S2 OR S3 OR S4 OR S5 OR S6 OR S7 OR S8 OR S9 OR S10  S12 "motivational interview*" OR "motivation interview*"  S13 S1 AND S11 AND S12  S14 adult OR adults  S15 S13 AND S14  S16 child* OR adolescen* OR youth  S17 S13 NOT S16  S18 S15 OR S17  S19 rct OR rcts OR "randomized controlled" OR "randomised controlled" OR "random control" OR "randomized trial*" OR "randomised trial*" OR "random allocation" OR randomly OR randomisation* OR randomization* OR "clinical trial*" OR "controlled trial*" OR "control trial*" OR "double blind*" OR "double mask*" OR placebo*  S20 S18 AND S19  S21 S20 AND limit to English |
| Cochrane Library |
| #1 depress*:ti,ab,kw  #2 (intervention* OR therap* OR treatment*):ti,ab,kw  #3 (life NEXT style* OR lifestyle*):ti,ab,kw  #4 (exercise* OR sport* OR physical NEXT activit* OR fitness OR walking OR jogging OR jogger* OR running OR runner* OR gym OR gyms OR gymnastic* OR swim* OR skiing OR skier* OR weight NEXT lift* OR skating OR hockey OR football OR soccer OR basketball OR volleyball):ti,ab,kw  #5 (diet* OR food* OR nutrit* OR nutrient* OR eating OR meal* OR breakfast* OR brunch* OR lunch* OR dinner* OR supper*):ti,ab,kw  #6 (sleep* OR dyssomnia* OR parasomnia* OR somnole*):ti,ab,kw  #7 (smok* OR tobacco OR cigar* OR e-cig* OR snuff* OR nicotine NEXT addict*):ti,ab,kw  #8 ("quality of life" OR "life quality" OR "body weight" OR "body mass index" OR bmi OR "waist circumference" OR "blood pressure" OR hypertens* OR (lipid* NEAR/2 blood) OR (blood NEAR/2 glucose)):ti,ab,kw  #9 (stress* OR distress* OR anxiet*):ti,ab,kw  #10 (adherence OR compliance OR nonadherence OR noncompliance OR acceptance OR satisfaction OR refus* OR negative OR adverse OR worsen*):ti,ab,kw  #11 #2 OR #3 OR #4 OR #5 OR #6 OR #7 OR #8 OR #9 OR #10  #12 (motivational NEXT interview* OR motivation NEXT interview*):ti,ab,kw  #13 #1 AND #11 AND #12  #14 (adult OR adults):ti,ab,kw  #15 #13 AND #14  #16 (child* OR adolescen* OR youth):ti,ab,kw  #17 #13 NOT #16  #18 #15 OR #17 |

**Supplemental eTable 3.** Excluded eligible studies.

| First author, year, country | Population | Title | Reason for exclusion |
| --- | --- | --- | --- |
| Ismail et al. 2010, United Kingdom  (2) | 18-65 years, diabetes | A randomized controlled trial of cognitive behaviour therapy and motivational interviewing for people with type 2 diabetes mellitus with persistent sub-optimal glycaemic control: a Diabetes and Psychological Therapies (ADaPT) study. | Missing outcome data |
| Ismail et al. 2018, United Kingdom  (3) | 18-79 years, type 2 diabetes | Nurse-led psychological intervention for type 2 diabetes: a cluster randomized controlled trial (Diabetes-6 study) in primary care | Missing outcome data |

**Supplemental eTable 4.** The details of interventions and funding.

| Authors, year,country | Details of the interventions | Training in MI | Funding |
| --- | --- | --- | --- |
| Glasgow et al. 2006, USA  (4) | 1. Computer-assisted self-management assessment, 2. tailored self-management intervention, 3. approx. 1 week and 1 month after the first visit, a follow-up call (10 - 15 min) from their health coach to review their goals, barriers, and strategies, and reinforce or revise their plan as appropriate, 4. a tailored health newsletter was mailed approx. 6 weeks after the first visit. Health coaches answered any questions and helped to ensure that the final plan was appropriate for the individual. | Role play, hands-on practice, and exposure to motivational interviewing techniques using training videotapes created by Miller and Rollnick | National Institute of Diabetes & Digestive & Kidney Diseases |
| Katon et al. 2010,  USA  (5) | 1. The Depression Helpbook, 2. a video compact disk on depression care, 3. a booklet and other materials on chronic disease managements, 4. self-monitoring devices, 5. structured visits every 2 to 3 weeks to a primary care clinic, and 6. follow-up telephone calls every 4 weeks on the maintenance phase, for 12 months. | 2-day training course on depression management, behavioral strategies, and glycemic, blood pressure, and lipid control, and weekly supervision | Services Division of the National Institute of Mental Health |
| Van derWulp et al. 2012, Netherlands  (6) | The expert peer patients conducted 3 home visits (duration approx. 1 h) in 3 months + 3 phone calls within 2 weeks after each visit. | Three training sessions, each lasting 3.5 h. The basic principles of motivational interviewing were taught (how to support self-efficacy, coping with resistance, showing empathy, and exploring discrepancies). | Dutch Diabetes Research Foundation |
| Gabbay et al. 2013, USA  (7) | Individual meetings in a primary care clinic with nurse case managers at baseline, 2 weeks, 6 weeks, 3 months, 6 months, 12 months, and at least every 6 months thereafter (approx. 1 h/visit). Possibility to contact nurse case managers by phone and e-mail in between visits when appropriate | 80 h training (classroom lectures, role plays, videoconferences, journal article reviews, attending conferences and lectures, and mock interviews with standardized patients) | National Institutes of Health, and National Institute of Diabetes and Digestive and Kidney Diseases |
| Holmen et al. 2014, Norway  (8) | The participants received 5 telephone calls from the nurse during the first 4 months, approx. 20 min / call. A mobile phone with the Few Touch application for self-management (diabetes diary, a blood glucose data management system, food habit registration system, a physical activity registration system, a personal goal-setting system, and a general diabetes information look-up system) and the possibility to contact the diabetes specialist nurse via a secured text messaging system. | Not reported | 1. the EU through the ICT Policy Support Programme as part of the Competitiveness and Innovation  Framework Programme, 2. the Norwegian Research Council, 3. the Health Authorities of Northern Norway  , 4. the NorwegianCentre of Integrated Care and Telemedicine at the University Hospital of North-Norway  , 5. the Oslo and Akershus University College, 6. the Akershus University Hospital, and 7. the Norwegian Diabetes Association. |
| Huang et al. 2016, Taiwan  (9) | A total of 12 weekly sessions over 3 months, each lasting 80 min, in two 15 - 16 patient groups: 4 sessions of motivational enhancement therapy over a period of 1 month, followed by 8 sessions of Cognitive behavioral therapy for two additional months. | Not reported | the National Science Council |
| Swoboda et al. 2017, USA  (10) | 1. One baseline in-person goal setting and decision coaching session followed by 7 biweekly coaching calls, 2. Goals and action plans were mailed to participants after each coaching session. | Not reported | The Ohio State University Department of Human Sciences |
| Azami et al. 2018, Iran  (11) | 1. A detailed information booklet on conducting self-management given at the beginning of program, 2. four 10 min weekly movie clips (based on booklet) for the first 4 weeks, 3. four weekly, group-based educational sessions (approx. 120 min, maximum of 10 participants in each session), 4. follow-up calls weekly two months after the end of the group sessions (approx. 15-20 min). | Not reported | University Putra Malaysia, Ilam University of  Medical Sciences, Ilam, Iran. |
| Döbler et al. 2018,  Germany  (12) | 1. A 1-hr face-to-face introductory session at the end of the rehabilitation stay to assess participants' individual behavior, 2. individualized action and coping plan for transferring lifestyle changes into the patient's daily routine, 3. the study counselors called patients monthly for 12 months | 3-day training course | The Rehabilitations-Forschungsnetzwerk of the  Deutsche Rentenversicherung Rheinland |
| Ali et al. 2020, India  (13) | Collaborative care model (a care coordinator, psychiatrist, diabetologist, diabetes physicians)  1. Care coordinators: every 2-4 weeks phone or in-person contact with patients, 2. during contacts, care coordinators monitored depressive symptoms, reviewed glucose and/or blood pressure logs, counseling aimed at achieving individualized treatment goals,  3. A decision support electronic health record system based on treatment guidelines to support diabetes physicians in their clinical decision-making and/or timely modification of evidence-based behavioral or pharmacotherapies for depression, glucose, blood pressure, and lipid management. | 3-day training included 60 min session on motivational interviewing (didactics, demonstrations, role plays, feedback) | National Institute of Mental Health |
| Young et al. 2020,  USA (14) | Nurse health coaching and mHealth technology to track patient-generated health data (steps taken, distance walked, active minutes, heart rate, sleeping hours; iPod Touch [MyFitnessPal] to log and track nutritional consumption) and integrate these data into the electronic health records. Coaching included an in-person orientation and individual counseling sessions via phone calls in every 2 weeks for 3 months (6 contacts total). | Core training in motivational interviewing-based coaching, health coaching competency evaluated through MITI | Patient-Centered Outcomes Research Institute |

**Supplemental eFigure 1.** Risk of bias across studies.

**

**

**Supplemental eFigure 2**. Forest plot for 10 studies combined.


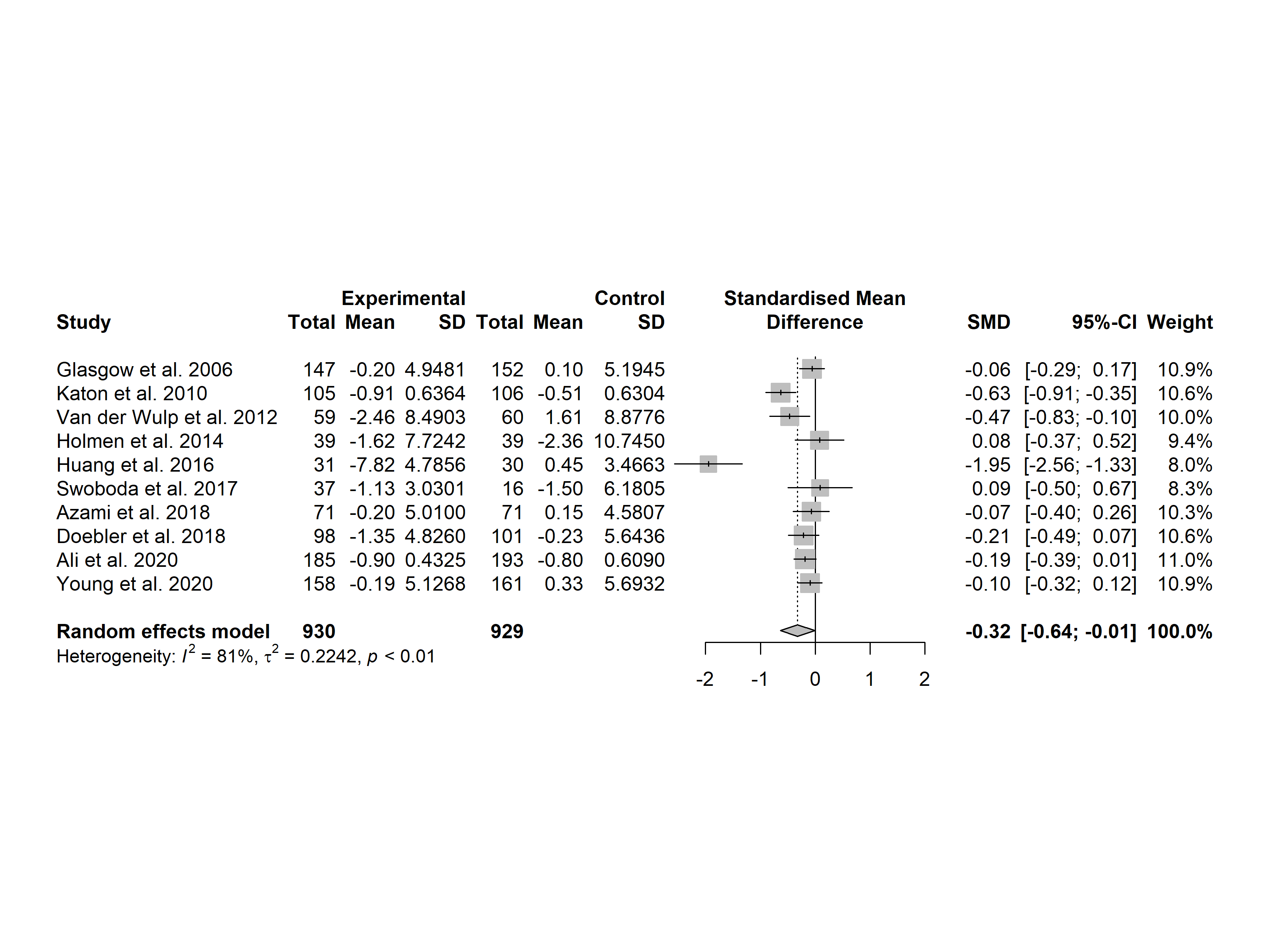


**Supplemental eFigure 3**. Funnel plot for 10 studies combined.


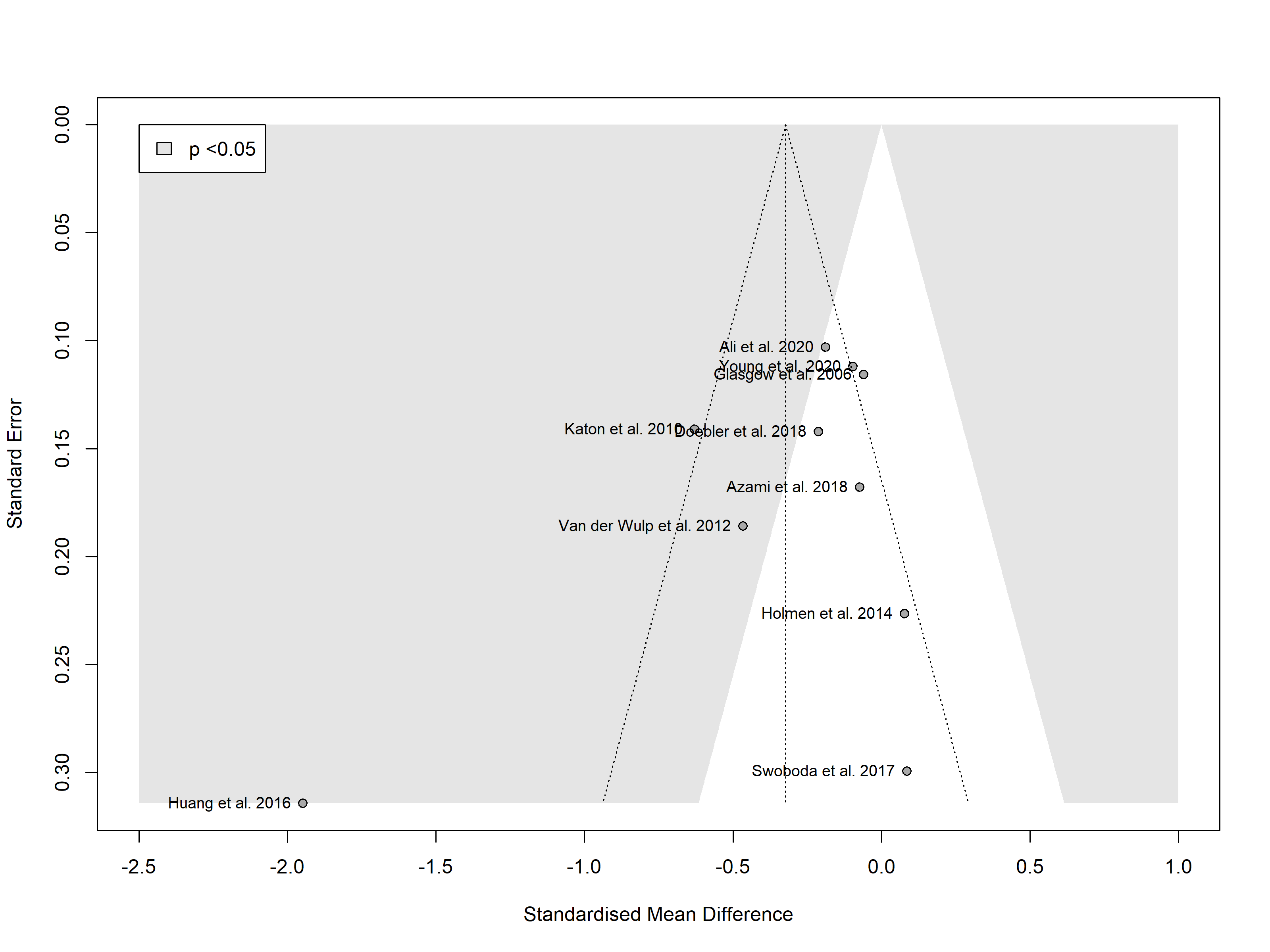


**Supplemental eFigure 4.** Funnel plot for 9 studies combined.


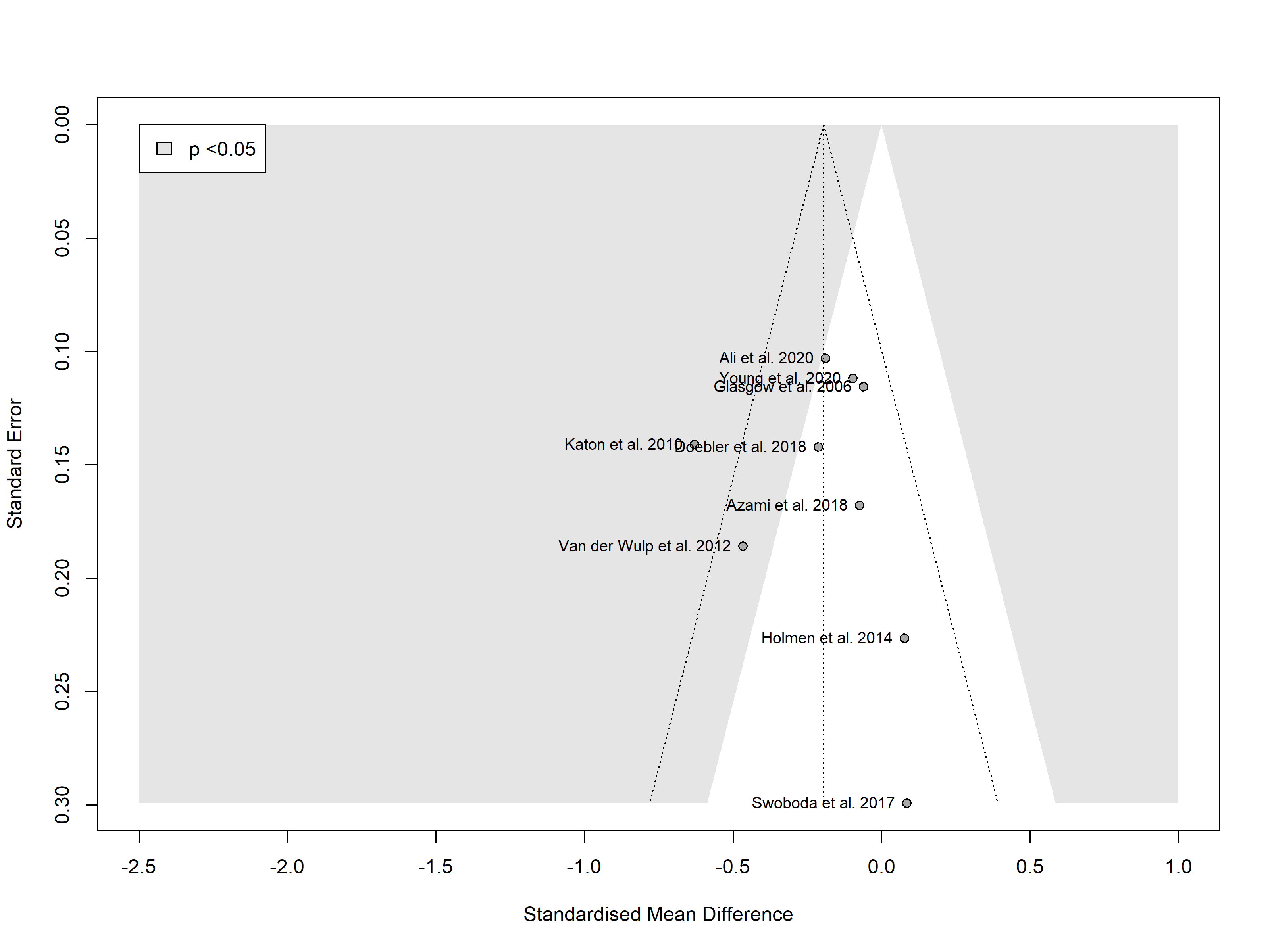


**Supplemental eFigure 5.** Forest plot for subgroup meta-analysis of 9 studies combined.


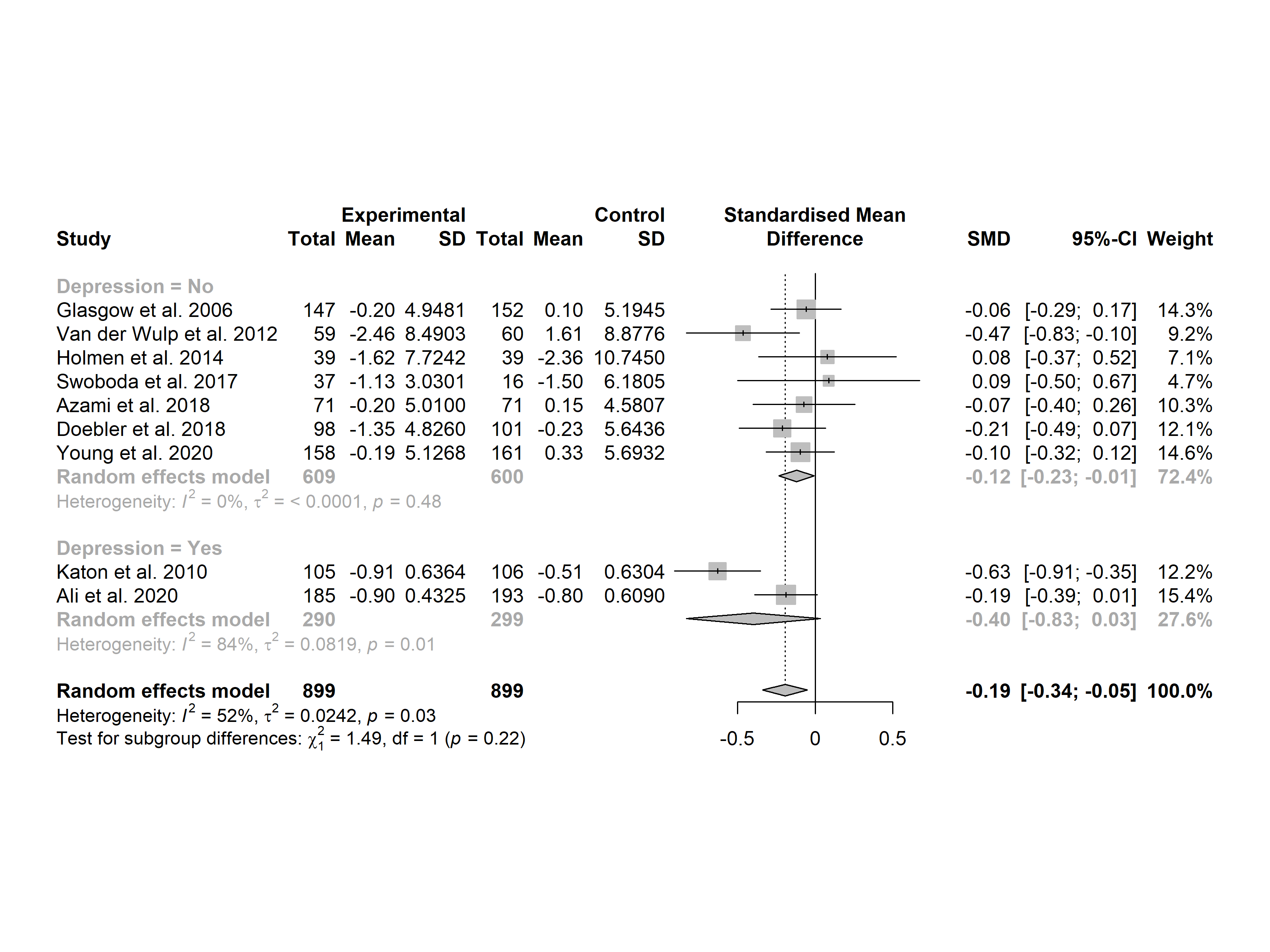


*Depression=Yes (Populations with elevated depressive symptoms)*

1. Page MJ, McKenzie JE, Bossuyt PM, Boutron I, Hoffmann TC, Mulrow CD, Shamseer L, Tetzlaff JM, Akl EA, Brennan SE, Chou R, Glanville J, Grimshaw JM, Hróbjartsson A, Lalu MM, Li T, Loder EW, Mayo-Wilson E, McDonald S, McGuinness LA, Stewart LA, Thomas J, Tricco AC, Welch VA, Whiting P, Moher D. The PRISMA 2020 statement: an updated guideline for reporting systematic reviews. BMJ 2021;372 :n71.

2. Ismail K, Maissi E, Thomas S, Chalder T, Schmidt U, Bartlett J, Patel A, Dickens C, Creed F, Treasure J. A randomised controlled trial of cognitive behaviour therapy and motivational interviewing for people with Type 1 diabetes mellitus with persistent sub-optimal glycaemic control: A Diabetes and Psychological Therapies (ADaPT) study. Health Technol.Assess. 2010;14 :1-218.

3. Ismail K, Winkley K, de Zoysa N, Patel A, Heslin M, Graves H, Thomas S, Stringer D, Stahl D, Amiel SA. Nurse-led psychological intervention for type 2 diabetes: a cluster randomised controlled trial (Diabetes-6 study) in primary care. Br.J.Gen.Pract. 2018;68 :e531-e540.

4. Glasgow RE, Nutting PA, Toobert DJ, King DK, Strycker LA, Jex M, O'Neill C, Whitesides H, Merenich J. Effects of a brief computer-assisted diabetes self-management intervention on dietary, biological and quality-of-life outcomes. Chronic Illn 2006;2 :27-38.

5. Katon WJ, Lin EHB, Von Korff M, Ciechanowski P, Ludman EJ, Young B, Peterson D, Rutter CM, McGregor M, McCulloch D. Collaborative care for patients with depression and chronic illnesses. N Engl J Med 2010;363 :2611-2620.

6. van der Wulp I, de Leeuw, J. R. J., Gorter KJ, Rutten, G. E. H. M. Effectiveness of peer-led self-management coaching for patients recently diagnosed with Type 2 diabetes mellitus in primary care: a randomized controlled trial. Diabet Med 2012;29 :390.

7. Gabbay RA, Añel-Tiangco RM, Dellasega C, Mauger DT, Adelman A, Van Horn, D. H. A. Diabetes nurse case management and motivational interviewing for change (DYNAMIC): Results of a 2-year randomized controlled pragmatic trial. Journal of Diabetes 2013;5 :349-357.

8. Holmen H, Torbjørnsen A, Wahl AK, Jenum AK, Småstuen MC, Arsand E, Ribu L. A Mobile Health Intervention for Self-Management and Lifestyle Change for Persons With Type 2 Diabetes, Part 2: One-Year Results From the Norwegian Randomized Controlled Trial RENEWING HEALTH. JMIR Mhealth Uhealth 2014;2 :e57.

9. Huang C, Lai H, Chen C, Lu Y, Li S, Wang L, Su Y. Effects of motivational enhancement therapy plus cognitive behaviour therapy on depressive symptoms and health-related quality of life in adults with type II diabetes mellitus: A randomised controlled trial. Qual.Life Res. 2016;25 :1275-1283.

10. Swoboda CM, Miller CK, Wills CE. Impact of a goal setting and decision support telephone coaching intervention on diet, psychosocial, and decision outcomes among people with type 2 diabetes. Patient Educ.Couns. 2017;100 :1367-1373.

11. Azami G, Soh KL, Sazlina SG, Salmiah MS, Aazami S, Mozafari M, Taghinejad H. Effect of a Nurse-Led Diabetes Self-Management Education Program on Glycosylated Hemoglobin among Adults with Type 2 Diabetes. J Diabetes Res 2018;2018 :4930157.

12. Döbler A, Belnap BH, Pollmann H, Farin E, Raspe H, Mittag O. Telephone-delivered lifestyle support with action planning and motivational interviewing techniques to improve rehabilitation outcomes. Rehabilitation Psychology 2018;63 :170-181.

13. Ali MK, Chwastiak L, Poongothai S, Emmert-Fees K, Patel SA, Anjana RM, Sagar R, Shankar R, Sridhar GR, Kosuri M, Sosale AR, Sosale B, Rao D, Tandon N, Narayan KMV, Mohan V. Effect of a Collaborative Care Model on Depressive Symptoms and Glycated Hemoglobin, Blood Pressure, and Serum Cholesterol among Patients with Depression and Diabetes in India: The INDEPENDENT Randomized Clinical Trial. JAMA - Journal of the American Medical Association 2020;324 :651-662.

14. Young HM, Miyamoto S, Dharmar M, Tang-Feldman Y. Nurse Coaching and Mobile Health Compared With Usual Care to Improve Diabetes Self-Efficacy for Persons With Type 2 Diabetes: Randomized Controlled Trial. JMIR Mhealth Uhealth 2020;8 :e16665.
